# Observation of uniaxial anisotropy along the [100] direction in crystalline Fe film

**DOI:** 10.1038/srep17761

**Published:** 2015-12-04

**Authors:** Seul-Ki Bac, Hakjoon Lee, Sangyoep Lee, Seonghoon Choi, Taehee Yoo, Sanghoon Lee, X. Liu, J. K. Furdyna

**Affiliations:** 1 Physics Department, Korea University, Seoul 136-701, Korea; 2 Physics Department, University of Notre Dame, Notre Dame, IN 46556, USA

## Abstract

We report an observation of uniaxial magnetic anisotropy along the [100] crystallographic direction in crystalline Fe film grown on Ge buffers deposited on a (001) GaAs substrate. As expected, planar Hall resistance (PHR) measurements reveal the presence of four in-plane magnetic easy axes, indicating the dominance of the 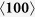 cubic anisotropy in the film. However, systematic mapping of the PHR hysteresis loops observed during magnetization reversal at different field orientations shows that the easy axes along the 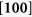 and 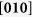 are not equivalent. Such breaking of the cubic symmetry can only be ascribed to the presence of uniaxial anisotropy along the 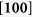 direction of the Fe film. Analysis of the PHR data measured as a function of orientation of the applied magnetic field allowed us to quantify the magnitude of this 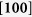 uniaxial anisotropy field as 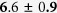 Oe. Although this value is only 1.5% of cubic anisotropy field, its presence significantly changes the process of magnetization reversal, revealing the important role of the 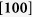 uniaxial anisotropy in Fe films. Breaking of the cubic symmetry in the Fe film deposited on a Ge buffer is surprising, and we discuss possible reason for this unexpected behavior.

The iron films are broadly used in a wide range of the magnetic devices. Recently, Fe films interfaced with semiconductors materials have attracted particular attention owing to the possibility of new functionalities that arise from such hybridization of ferromagnetic metals and semiconductors^1^,^2^,^3^. Successful growth of high-quality crystalline Fe film on various semiconductor surfaces, such as GaAs^4^,^5^,^6^, ZnSe^6^,^7^, MgO^8^,^9^,^10^, and Ge^6^,^11^,^12^, by an epitaxial technique further adds to the interest in Fe/semiconductor combinations^13^.

The magnetic anisotropy is a critically important property of a ferromagnetic film, since it determines the easy magnetization directions of the film at low magnetic field or in its absence. The magnetic anisotropy of crystalline Fe film has been investigated quite extensively by many experimental techniques, revealing its complex nature that arises from the simultaneous presence of the cubic magnetic anisotropy along the 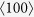 and the uniaxial anisotropy along one of the 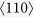 directions^14^,^15^. While the former originates from the cubic crystal structure of Fe, the latter is related to surface reconstruction of the substrate, which determines the specific symmetry of bond alignments on surfaces onto which Fe atoms are epitaxially deposited^16^,^17^,^18^,^19^,^20^. So far, only these two types of anisotropies have been experimentally detected in crystalline Fe films grown on semiconductor substrates^6^,^19^,^21^,^22^,^23^,^24^,^25^. Even though detailed discussion for the uniaxial anisotropy of Fe film was given in Ref. [16] and [19], it was only about the [110] uniaxial anisotropy. The [100] uniaxial anisotropy, which is the major issue of this paper, has never been observed, nor has it even been discussed in those previous studies.

In this paper, we report the observation of a new type of uniaxial anisotropy, associated with the in-plane 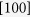 crystallographic direction in the Fe film. To investigate magnetic anisotropy of the Fe film, we make use of the planar Hall effect (PHE), which arises from the tensor character of the anisotropic magnetoresistance^26^,^27^. Detection of this uniaxial anisotropy is possible due to the fact that in PHE measurements even very small contributions of a uniaxial anisotropy to the reorientation of magnetization between magnetic easy axes lead to conspicuous asymmetric behavior of the hysteresis loops observed by planar Hall resistance (PHR), as discussed below.

## Experiments

**Figure 1:**
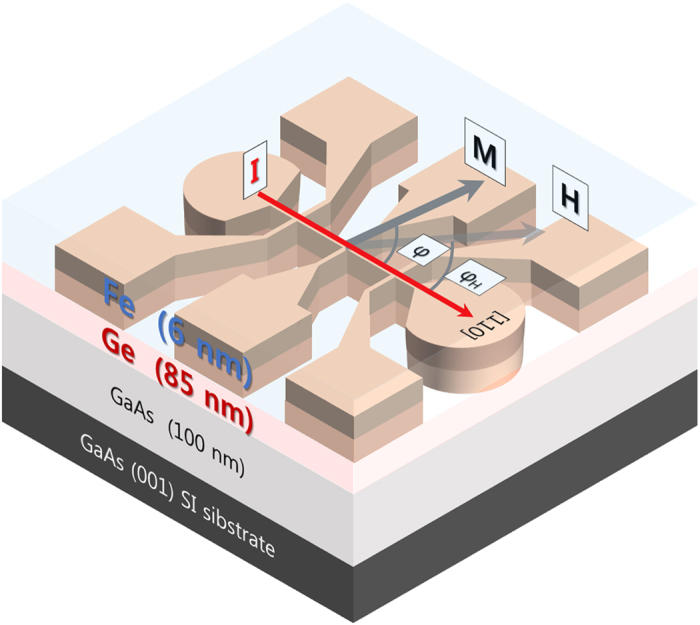
Schematic diagram of the Hall device for PHR measurement patterned on Fe film grown on Ge buffer. Directions of the current ***I***, magnetization ***M***, and external field ***H*** are shown by arrows.

The Fe film used in this investigation was grown on (001) GaAs substrate by molecular beam epitaxy (MBE). Prior to the Fe deposition, an 85 nm Ge buffer layer was deposited on the GaAs substrate. The Fe layer was then directly grown on the Ge buffer to a thickness of 6 nm. The Fe layer was finally covered by a thin layer of Au (2 nm) to protect the Fe layer from oxidation. The choice of Ge buffer was dictated by the fact that in this situation any uniaxial anisotropy in Fe films associated with the 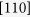 direction (i.e., any anisotropy originating from surface reconstruction during deposition) is completely eliminated^6^, thus enabling one to clearly distinguish possible new deviations from a fully four-fold symmetry in the film plane. To investigate the magnetic anisotropy properties of the Fe film obtained in this way by PHE experiments, a 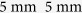 piece was cut from the wafer, and a Hall device was fabricated by photolithography and dry etching. The Hall device was in the form of a 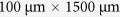 rectangle, two current leads and four Hall voltage leads. A schematic diagram for the Hall device is shown in Fig. 1, with the directions of the current, the magnetization, and the external field indicated by arrows.

The PHR measurements were performed using a sample holder designed so as to allow a magnetic field to be applied at arbitrary direction in the plane of the sample. The electromagnet used for this purpose was mounted on a rotatable table, so that the field could either be swept along an arbitrary fixed direction, or could be continuously rotated in the film plane with a fixed magnitude. The azimuthal angles 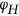 and 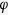, indicating the direction of the external field and the magnetization of the Fe films, respectively, were measured counterclockwise from the 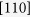 crystallographic direction, as shown in Fig. 1. The direction of the current in the Hall device was 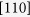. The PHR was measured during the magnetization reversal, which was performed in one of two ways: either the magnetization was reversed by sweeping the magnetic field intensity with the field applied at a series of fixed angles 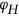; or a field with a fixed magnitude was rotated over 360° in the clockwise (CW) or counterclockwise (CCW) direction. The PHR measurements under both forms of magnetization reversal were carried out at 3 K.

## Results and Discussion

**Figure 2:**
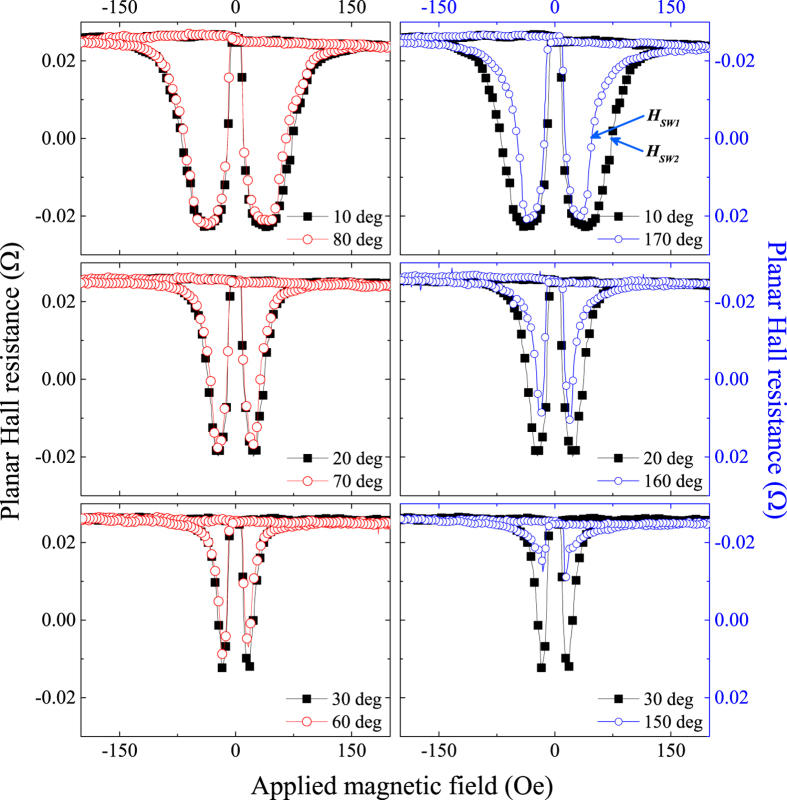
PHR data obtained by sweeping the field between −200 *Oe* and 200 *Oe* at 3 K along the field directions oriented symmetrically on opposite sides of [010] (left column) and to 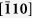 (right column) crystallographic directions. The data in the left-hand panels closely overlap, indicating the same energetic processes for sweeps along direction symmetric with respect to the 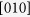. However, the data in the right-hand panels show clear difference in switching fields (at positions marked 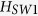 and 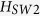) and in amplitude, indicate that sweeps along directions symmetric with respect to the 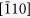 are energetically not equivalent, i.e., that magnetic anisotropy of the Fe films lacks four-fold symmetry.

Figure 2 shows PHR data taken by sweeping the field from −200 Oe to +200 Oe at different fixed field orientation 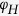. The two sets of data (open circles and solid squares) in the panels in the left column of the figure were obtained with field directions oriented symmetrically on opposite side of the 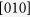 direction (i.e., 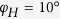 and 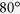), while those of the right column were obtained with field directions that are symmetric with respect to the 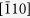 direction (i.e., 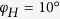 and 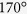). Note that in the right column of Fig. 2 the vertical axis has been inverted for the open symbols (as marked on the right side of the figure), so as to facilitate comparison of the PHR data obtained at 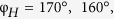 and 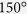 with those in the left-hand panels obtained at 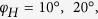 and 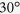.

**Figure 3:**
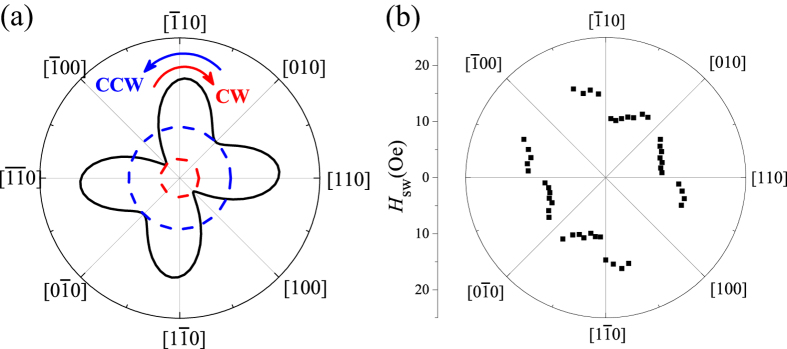
(**a**) Schematic polar plot of the magnetic free energy in the layer plane with dominant cubic symmetry and a small admixture of uniaxial symmetry associated with one of the 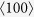 crystallographic axes. The magnitude of the uniaxial energy is exaggerated for clarity. (**b**) Polar plot of switching field 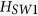 observed in field sweeps of PHR taken at 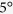 increments in the plane of the Fe film. Note that absence of four-fold symmetry expected in a purely cubic material, indicating a presence of a uniaxial component in the magnetic anisotropy in the system.

As is well known, the “jumps” in the value of PHR seen in Fig. 2 occur at fields at which the magnetization switches from one in-plane easy axis to another during the field sweep^28^,^29^. The curves in each panel in the left column (i.e., the PHR curves measured with fields oriented symmetrically around the 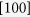 direction) closely overlap. However, the PHR curves in each of the panels in the right column show clear differences in switching fields, marked as 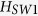 and 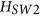. We attribute this behavior to the presence of uniaxial anisotropy associated with the 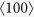 directions. This type of anisotropy can be schematically represented by the polar plot of the magnetic free energy shown in Fig. 3(a).

As an example of the effect of such anisotropy on PHR, consider the case where a field applied along 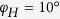 is swept from positive to negative direction and back. As the field approaches zero, the magnetization is aligned with the 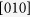 easy axis, and remains so aligned when the field changes sign. The negative field now has two components, along 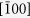 and 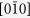, causing the free energy minima along both these directions to decrease. Since the 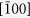 easy axis is adjacent to the original direction of the magnetization, at some value of negative field the magnetization switches to that axis, resulting the first “jump” of PHR seen at low negative field in Fig. 2. As the negative field increases further, the increasing field component along the adjacent deeper easy axis 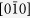 will eventually drive the magnetization to that direction, resulting in the second “jump” in PHR. Note, however, that in the presence of uniaxial anisotropy the switching process is not energetically identical in these two steps, i.e., the transition from 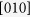 to 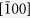 is “easier” because the magnetization starts from a higher energy to a lower energy, as seen in Fig. 3(a).

It is easy to see that the process of magnetization reversal for a field oriented symmetrically on the other side of the 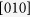 aixs (i.e., at 80°) is energetically the same: when the field is reversed, the magnetization first reorients (now turning CW) from the higher 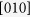 minimum to the deeper minimum at 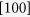, and then to 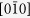. The energetically identical path for field orientations along 10° and 80° means that the fields at which the two jumps in PHR observed at these orientations will the same for both these orientations, as is indeed seen in the upper left-hand panel of Fig. 2.

The situation is, however, different when we compare the process of field reversal for field orientations that are symmetric about one of the hard axes, e.g., 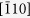. As an example, we compare the sweep of the field along 10° just discussed with that observed for 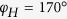. In the latter case, as the field approaches zero, the magnetization is initially oriented along the deep 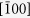 minimum. As the field reverses, it acquires components along the 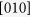 and 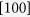 directions, causing the magnetization to first rotate to the closer 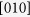 direction, and then to 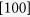. Note that now the first rotation is “harder”, involving a reorientation from a deeper to more shallow minimum, so that the field required to achieve the first reorientation (designated as 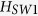) will be higher than in the case for the 10° field sweep. The second reorientation, on the other hand, corresponding to the second switching field in PHR (designated as 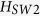), involves a transition from a shallow to a deep minimum, and thus will occur at a lower field than was the case for the 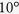 sweep, exactly as is seen in the upper-right panel of Fig. 2. It is easy to show that, if the polar plot in Fig. 3(a) were four-fold symmetric (i.e., in the absence of the uniaxial 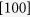 anisotropy), the PHR data in the right-hand panels in Fig. 2 would coincide.

To obtain a detailed mapping of the consequences of the 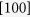 magnetic anisotropy, we performed field sweep measurements of PHR with 5° increments of field orientation over 360°. The switching fields 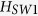 obtained from such field sweeps are shown as a polar plot in Fig. 3(b). The pattern of 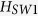 in the figure clearly shows a significant distortion from the in-plane four-fold symmetry, indicating the presence of an anisotropy other than the cubic in the Fe film, associated with the 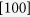 direction. We should recall here that occurrence of the uniaxial anisotropy associated with one of the 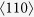 directions is known to occur in cubic systems such as Fe and GaMnAs when they are deposited on zinc-blende compound semiconductors such as GaAs or ZnSe, and is ascribed to the asymmetry of surface reconstruction during such epitaxial deposition^6^. In the present case that form of anisotropy has been eliminated by growing the Fe films on a Ge buffer. The uniaxial anisotropy associated with the 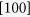 crystallographic direction, observed in the present investigation, is thus an entirely new and unexpected effect.

**Figure 4:**
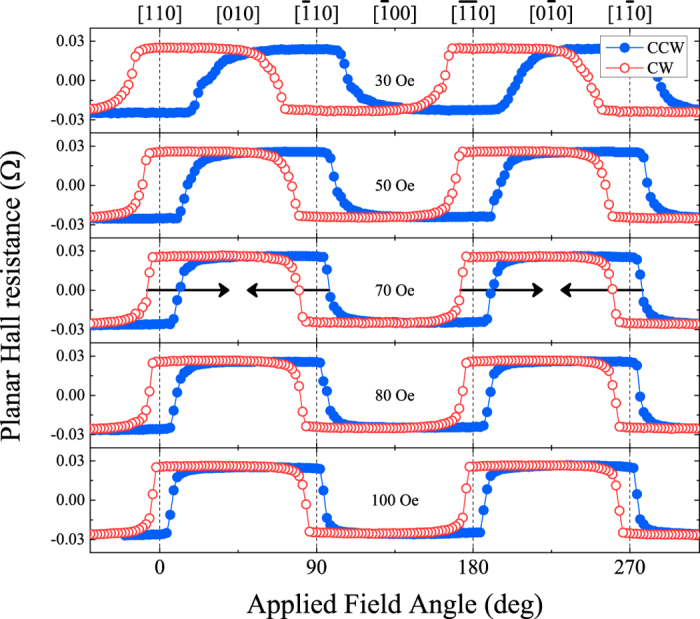
Angular dependence of PHR measured in the plane of the Fe film at 3 K using a rotating field. The open (red) and solid (blue) symbols show data taken with field rotating in the CW and CCW directions, respectively. The centers of the hysteresis loops around the 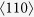 directions show shifts toward the 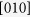 and the 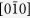 directions, as marked with black arrows in the central panel. The PHR data in each panel was taken with different field strength as written in the respective panel.

The presence of such uniaxial anisotropy is further confirmed by PHR measurements carried out by rotating the external field direction 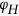 in the sample plane while the field strength was kept constant. The angular dependences of the PHR data obtained at 3 K using various field strengths are plotted in Fig. 4, where the open (red) and solid (blue) values are obtained by rotations of the applied field in CW and CCW direction, respectively. The data show clear hysteresis loops across all four 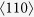 energy barriers shown in Fig. 3(a). Such angular hysteresis loops in this experiment arise as follows: when a weak field rotates CW across, e.g., the 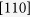 energy barrier in Fig. 3(a), it must move well into the 4^th^ quadrant before the magnetization switches from the 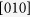 to the 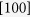 direction; and, similarly, when the field rotates CCW across this barrier, it must be in the 1^st^ quadrant to achieve reorientation from 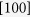 to 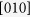. The existence of four such angular hysteresis loops clearly indicate that the Fe film has four in-plane magnetic easy axes near the 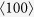 directions originating from the dominance of the cubic anisotropy, as seen in Fig. 3(a). The width of the hysteresis loops around the 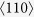 energy barriers are nearly the same for all barrier, indicating that there is no detectable the uniaxial anisotropy along the one of 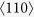 direction, such as is normally observed in the PHR measurements on Fe film grown on GaAs substrate^30^.

**Figure 5:**
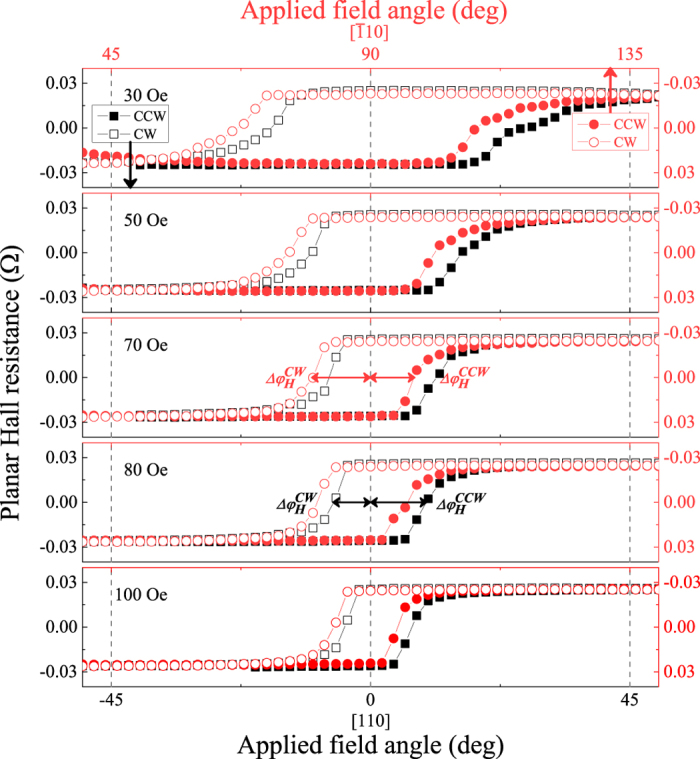
Plot of the hysteresis loops of PHR observed across the [110] (i.e., at 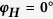, shown as empty symbols) and the 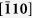 directions (i.e., at 90°, full symbols) observed by rotating the field with a series of fixed values. The circles and squares indicate CW and CCW rotation. The horizontal axis is adjusted to align 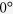 and 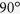 at the same position. The asymmetric shift of the switching angle 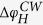 and 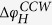 from the 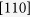 and the 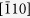 directions, and the shift of the hysteresis centers from those directions, is clearly seen in the plots.

**Figure 6:**
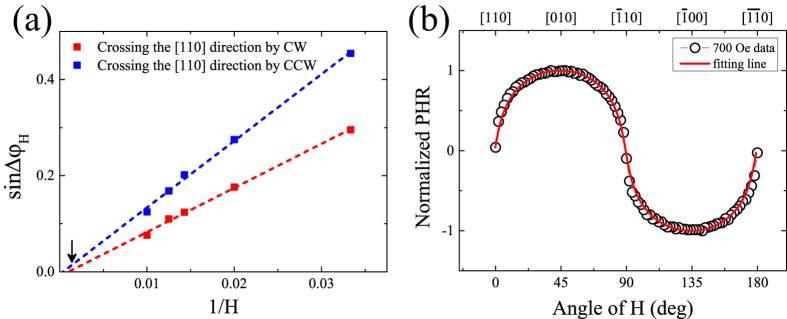
(**a**) Field dependences of the switching angle 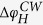 and 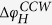 observed for the Fe film. The red and blue squares represent data for the 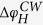 and 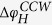 obtained from the hysteresis appeared at the 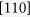 direction. The dotted lines are fitting results obtained using Eq. (2). (**b**) Angular dependence of PHR measured on the Fe film at 3 K with field strength of 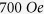. The circles are experimental; the solid line is the best fit obtained by using conditions for minimizing the magnetic free energy density given in Eq. (1).

A feature of key importance in the angle-dependent PHR data in Fig. 4 is the shift of the hysteresis loop centers toward the 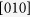 and 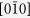 directions (and away from the 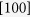 and 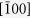), as indicated by the arrows in the third panels. To show this more clearly, we overlapped the PHR hysteresis around 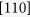 (0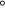, full black symbols) and 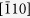 (90°, open red symbols) on an expanded scale in Fig. 5, where the circles and squares denote CW and CCW rotations. The figure clearly shows that the hysteresis loops around the 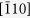 and the 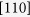 orientations are shifted opposite direction, indicates that the transitions of magnetization occurring over the 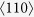 directions are different for the CW and CCW rotations. The origin of this difference can be seen from the following example. Referring to Fig. 3(a), we readily see that, in the CCW rotation, the field must rotate over a greater angle beyond the 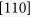 barrier in order to reorient the magnetization from its easy axis at 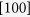 to 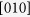 than the angle required for the CW-rotating field to achieve the 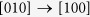 reorientation. The differences between the angles at which the magnetization switches measured from the 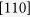 barrier are defined as switching angle 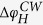 and 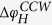 for CW and CCW rotations, respectively, and plotted in Fig. 6(a). This type of hysteresis shift in opposite direction across the two positions (i.e., at 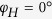 and 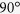) was also observed in GaMnAs film having a weak uniaxial anisotropy along the 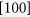 direction^31^,^32^.

The magnetization reversal results obtained by coherent rotation of magnetization using a strong magnetic field can be conveniently used to quantify the magnitude of magnetic anisotropy parameters of our Fe film by using magnetic free energy and Stoner-Wolfath model^33^. Since the uniaxial anisotropy is along the [100] direction, and there is no detectable uniaxial anisotropy associated with the 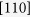 direction, the magnetic free energy for our Fe film can be expressed as^31^,^34^,^35^


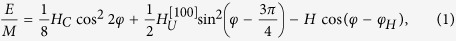


where 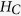 is the cubic anisotropy field, 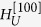 is the uniaxial anisotropy field associated with the 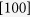 direction, 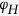 denotes the direction of the applied magnetic field, and 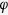 is the direction of the magnetization in the film. Since the magnetization of the film follows the free energy minima and the value of PHR depends directly on the direction of the magnetization, the angle dependence of PHR can be fitted by using the magnetic anisotropy fields as fitting parameters. This method of analysis is well described in the literatures^35^, and we adapted the same procedure to the present case.

We will first use the PHR data obtained with strong field to determine the magnitude of the cubic anisotropy of the film. We note from Fig. 6(a) that the switching angles 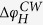 and 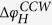 (and thus also the effect of the 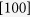 uniaxial anisotropy) decrease as the magnitude of the rotating field is increased, making PHR data obtained with higher fields particularly suitable for a “clean” determination of the cubic anisotropy parameters. For example, the values of 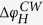 and 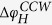 and their difference become nearly zero at 700 Oe as marked with an arrow in Fig. 6(a), indicating that the effect of 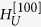 becomes negligible at that field. We therefore use the angular dependence of PHR taken with 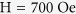 (plotted as open circles in Fig. 6(b)) and Eq. (1) without 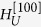 to obtain the cubic anisotropy field of the film. Fitting of the angular dependence of PHR data is shown as solid line in Fig. 6(b), giving the value of the cubic anisotropy field as 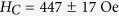.

The magnitude of the 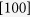 uniaxial anisotropy can be determined by analyzing the switching angles 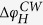 and 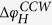 obtained at weak fields (in the range 30–100 Oe), shown in Fig. 6(a). The difference between 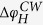 and 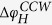 is caused by the difference in depths of magnetic free energy at the 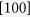 and the 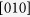 directions involved in the transition, as modeled by Cowburn *et al.* in ref. [36]. The magnetic field dependence of this switching angle can be described by^31^


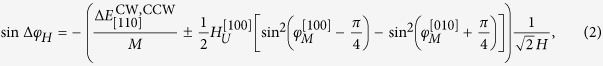


where 
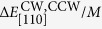
 are the differences in the magnetic free energy density between the above two energy minima, defined as 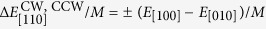, which can be obtained by analyzing the hysteresis^31^,^37^ at the 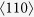 directions. Here the ± sign in the formula correspond to CW and CCW rotations. The experimental values of 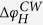 and 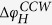 shown in Fig. 6(a) can be fitted using Eq. (2), where the only fitting parameter is 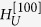. The detail process for this analysis is described in refs [31,37]. The best fitting of the PHR data with Eq. (2) (dashed lines in Fig. 6 (a)) provide the value of 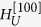 as 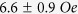.

The above quantitative analysis reveals that the magnitude of the 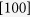 uniaxial anisotropy is only about 1.5% of the cubic anisotropy. Such small value of the 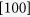 uniaxial anisotropy may be among the reasons why the existence of this anisotropy in Fe film has not been observed in earlier investigations. The present observations also serve to illustrate the advantages of PHR measurements, these sensitivity is sufficient to detect the effect caused by these small contributions of the uniaxial anisotropy to the magnetotransport experiments.

## Conclusion

We have investigated the magnetic anisotropy of the Fe film grown on GaAs substrate and the Ge buffer layer. Our PHR measurements clearly reveal the presence of the uniaxial anisotropy along the 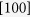 direction instead of the 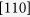-oriented anisotropy that is normally observed in Fe films grown directly on compound zinc-blende semiconductors such as GaAs of ZnSe. Even though the magnitude of the observed uniaxial anisotropy along the 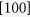 direction is only about 1.5% of the cubic anisotropy, it is seen to significantly affect the reorientation process of magnetization between its easy axes. The effect appears in the form of an angular pattern of switching fields that is strongly deformed from the four-fold symmetry expected in a cubic material, and in a strikingly asymmetric reorientation of magnetization observed as the applied field is rotated in opposite directions (i.e., CW and CCW) across the 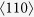 hard axes, as revealed in the angular dependence of PHR data.

Such 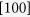 uniaxial anisotropy is not expected in the crystalline Fe films grown on a diamond-structured buffer, such as Ge, and we can only speculate about the causes responsible for this behavior. One possible origin of such 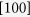-directed anisotropy is as follows. It is well known that an atomically-flat (001) Ge-surface on which the Fe layer is deposited is anisotropic because of the alignment of Ge-Ge bonds, such that the directions 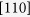 and 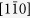 are not equivalent. However, in practice the MBE substrates (and therefore the buffers grown upon them) are not atomically flat, consisting of a series of atomic terraces, with Ge-bonds alternating between the 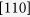 and 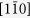 directions. This causes the net anisotropy associated with the 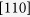 direction to disappear in the layer as a whole. However, in such slightly-vicinal surface the atomic terraces have a definite succession. We suggest that the combination of the two alternate 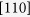 and 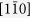 anisotropies of the succeeding individual terraces, together with the definite unidirectional sequence of the terraces, an lead to a resultant that makes the 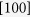 and 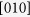 directions of the layer surface inequivalent. It is our hope that this report will stimulate further investigation aimed at identifying the origin of such uniaxial anisotropy along one of the 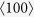 directions in Fe films.

## Additional Information

**How to cite this article**: Bac, S.-K. *et al.* Observation of uniaxial anisotropy along the [100] direction in crystalline Fe film. *Sci. Rep.* **5**, 17761; doi: 10.1038/srep17761 (2015).
